# Supplementary material for: Intratumor heterogeneity of EGFR expression mediates targeted therapy resistance and formation of drug tolerant microenvironment
Source: Nat Commun. 2025 Jan 2;16:28. doi: 10.1038/s41467-024-55378-5 (PMC11695629; doi:10.1038/s41467-024-55378-5)
Supplement: Supplementary file 1 — Supplementary Information File [file 41467_2024_55378_MOESM1_ESM.pdf]

Supplementary Figures

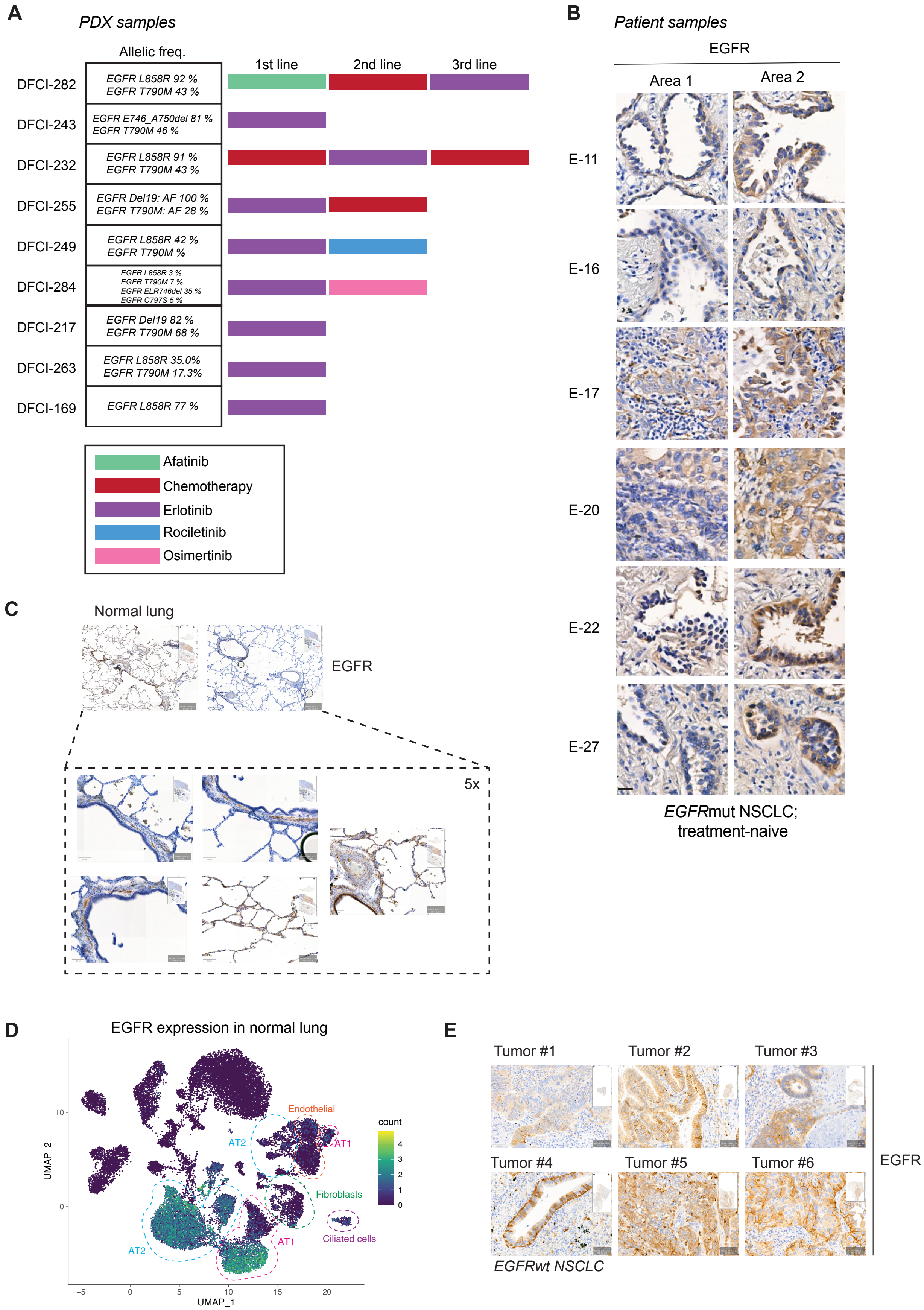

Supplementary Figure 1: (**A**) Treatment history for the PDX-grafted samples shown in Figure 1A. Tissue for PDX-grafting was collected in the end of the treatments. (**B**) Example images of EGFR staining heterogeneity in treatment-naïve NSCLC patient tissues from two different areas of the tumor. Scale bar is 50 µm. (**C**) EGFR expression in normal lung histotissues. Most of the staining was detected in bronchiole and alveoli of the lung. (**D**) Single-cell RNA sequencing analysis of EGFR expression in the normal lung. Most of the expression was detected in AT1 and AT2 cells, and heterogeneous expression could be observed within these populations. (**E**) EGFR expression in NSCLC tumors wild type for *EGFR*. Experiments shown in (**B, C, E**) were conducted once using patient samples.

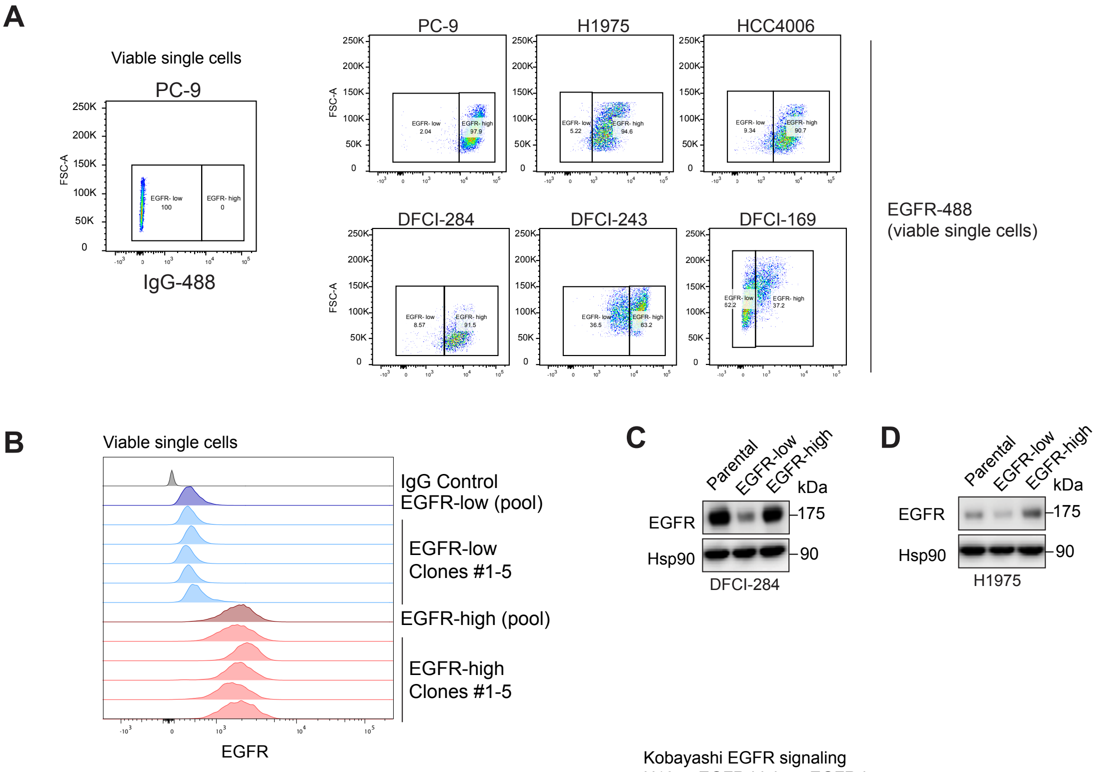

E

Cell line	*EGFR* copy number
PC-9 Parental	6.98
PC-9 EGFR-low	5.49
PC-9 EGFR-high	6.81

F

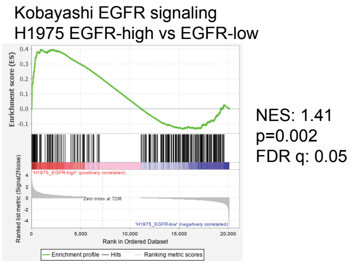

Supplementary Figure 2: (**A**) EGFR expression in *EGFR*-mutant non-small cell lung cancer cells. Upper row shows commercial cell lines, lower row shows cell lines established in-house. IgG-488 was used as a negative control. (**B**) EGFR expression shifts over time in clones originating from the EGFR-low or EGFR-high cells. IgG-488 was used as a negative control. (**C-D**) EGFR expression in DFCI-284 and H1975 parental, EGFR-low, and EGFR-high cell lines. Hsp90: Loading control. (**E**) Genomic EGFR copy number in PC-9 Parental, EGFR-low vs EGFR-high cells. Differences in copy number were non-significant (ANOVA, one-way). (**F**) EGFR gene signatures in H1975 EGFR-high vs EGFR-low cell lines.

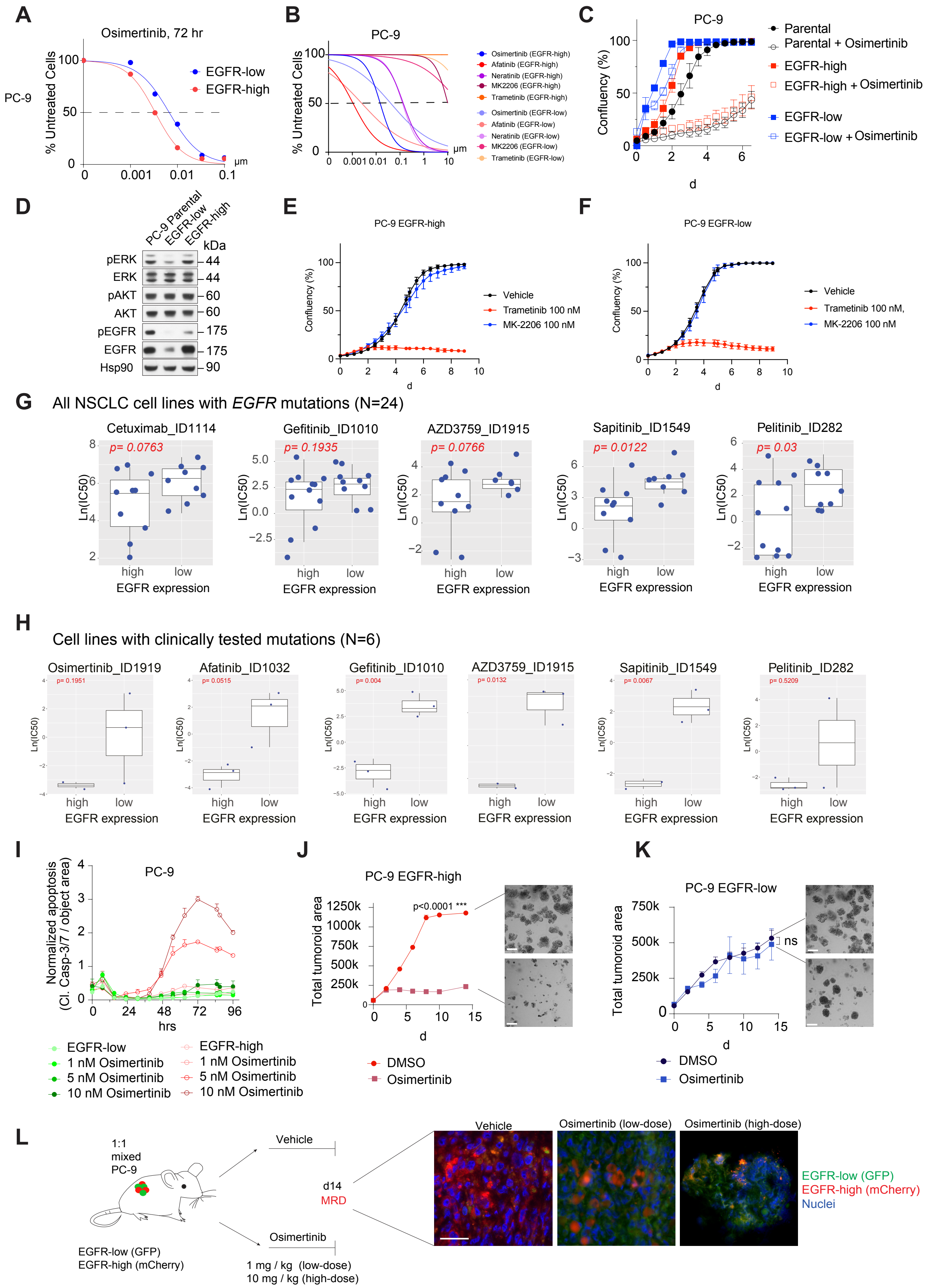
A
Osimertinib, 72 hr
PC-9
% Untreated Cells
EGFR-low
EGFR-high
μm
B
PC-9
% Untreated Cells
Osimertinib (EGFR-high)
Afatinib (EGFR-high)
Neratinib (EGFR-high)
MK2206 (EGFR-high)
Trametinib (EGFR-high)
Osimertinib (EGFR-low)
Afatinib (EGFR-low)
Neratinib (EGFR-low)
MK2206 (EGFR-low)
Trametinib (EGFR-low)
C
PC-9
Confluency (%)
d
Parental
Parental + Osimertinib
EGFR-high
EGFR-high + Osimertinib
EGFR-low
EGFR-low + Osimertinib
D
PC-9 Parental
EGFR-low
EGFR-high
kDa
pERK
ERK
pAKT
AKT
pEGFR
EGFR
Hsp90
E
PC-9 EGFR-high
Confluency (%)
Vehicle
Trametinib 100 nM
MK-2206 100 nM
F
PC-9 EGFR-low
Confluency (%)
Vehicle
Trametinib 100 nM,
MK-2206 100 nM
G
All NSCLC cell lines with EGFR mutations (N=24)
Cetuximab_ID1114
p= 0.0763
Gefitinib_ID1010
p= 0.1935
AZD3759_ID1915
p= 0.0766
Sapitinib_ID1549
p= 0.0122
Pelitinib_ID282
p= 0.03
Ln(IC50)
high
low
EGFR expression
H
Cell lines with clinically tested mutations (N=6)
Osimertinib_ID1919
p= 0.1951
Afatinib_ID1032
p= 0.0515
Gefitinib_ID1010
p= 0.004
AZD3759_ID1915
p= 0.0132
Sapitinib_ID1549
p= 0.0067
Pelitinib_ID282
p= 0.5209
Ln(IC50)
high
low
EGFR expression
I
PC-9
Normalized apoptosis
(Cl. Casp-3/7 / object area)
hrs
EGFR-low
1 nM Osimertinib
5 nM Osimertinib
10 nM Osimertinib
EGFR-high
1 nM Osimertinib
5 nM Osimertinib
10 nM Osimertinib
J
PC-9 EGFR-high
Total tumoroid area
p<0.0001 ***
d
DMSO
Osimertinib
K
PC-9 EGFR-low
Total tumoroid area
ns
d
DMSO
Osimertinib
L
1:1
mixed
PC-9
EGFR-low (GFP)
EGFR-high (mCherry)
Vehicle
Osimertinib
1 mg / kg (low-dose)
10 mg / kg (high-dose)
d14
MRD
Vehicle
Osimertinib (low-dose)
Osimertinib (high-dose)
EGFR-low (GFP)
EGFR-high (mCherry)
Nuclei

Supplementary Figure 3: (**A**) Osimertinib IC50 in PC-9 EGFR-low vs EGFR-high cells. Cells were treated with the drug for 72 hours. The experiment was biologically repeated 10 times. (**B**) IC-50 curves in PC-9 EGFR-low vs EGFR-high cells for Osimertinib (EGFRi), afatinib (EGFRi), neratinib (HER2i), MK2206 (AKTi) and Trametinib (MAPKi). Cells were treated with the drugs for 72 hours. The experiment was biologically repeated 8 times. (**C**) Osimertinib sensitivity is similar in PC-9 parental vs PC-9 EGFR-high cells. Cells were treated with 10 nM Osimertinib for 7 days. N = 3 biological replicates. (**D**) Western blot addressing MAPK/AKT signaling in PC-9 parental, EGFR-low, and EGFR-high cells. Hsp90: Loading control. (**E-F**) PC-9 EGFR-high vs PC-9 EGFR-low cell proliferation assays. Cells were treated with either vehicle DMSO, 100 nM MK-2206 (AKTi) or 100 nM trametinib (MEKi). N = 3 biological replicates. (**G**) Low EGFR RNA expression correlates with poor EGFR inhibitor response in *EGFR*-mutant NSCLC cell lines. Cell lines with any mutation affecting *EGFR* were included. CCLE database. (**H**) Low EGFR RNA expression correlates with poor EGFR inhibitor response in cell lines with clinically tested *EGFR* mutations. (**I**) PC-9 EGFR-high cells are more sensitive to osimertinib-induced apopotosis in a dose-dependent manner. (**J**) PC-9 EGFR-high cells are not able to form osimertinib resistance in 3D culture when cultured alone. Cells were treated with 10 nM Osimertinib for 15 days. Scale bar is 100 µm. Student's *t* test, unpaired. (**K**) PC-9 EGFR-high cells are highly resistant to osimertinib in 3D culture. Cells were treated with 10 nM Osimertinib for 15 days. Scale bar is 100 µm. Student's *t* test, unpaired. (**L**) EGFR-low cells are enriched in the MRD state. 1:1 mixed PC-9 EGFR-low : EGFR-high cells were grafted into mice and after tumor formation the mice were treated with either vehicle or Osimertinib (1 mg/kg or 10 mg/kg) for 14 days. After the treatment tumors were collected for imaging.

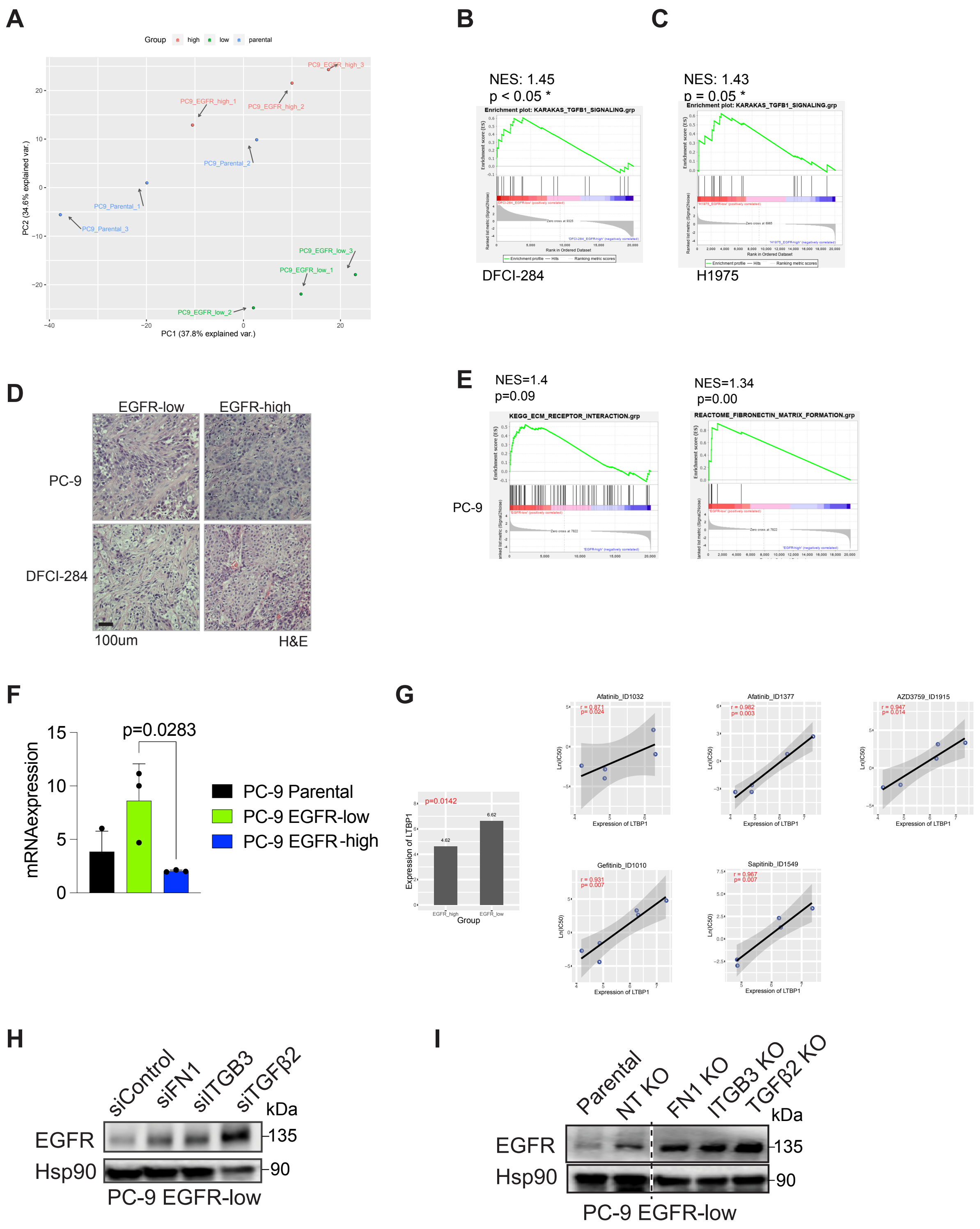

Supplementary Figure 4: (**A**) Principal component analysis from the replicate samples used in the RNA-sequencing. N= 3 biological replicates per sample. (**B**) TGFβ-signatures are upregulated in EGFR-low vs EGFR-high cell lines in both DFCI-284 and H1975 cells. (**D**) Xenograft tumors grafted from EGFR-low cells display more stromal phenotype than tumors grafted from EGFR-high tumors. Scale bar is 100 μm. The PDXs experiment was conducted once. (**E**) Gene signatures related to ECM-receptor interaction and fibronectin matrix formation are enriched in PC-9 EGFR-low cells. (**F**) *LTBP1* mRNA expression is significantly higher in PC EGFR-low cells compared to parental or EGFR-

high cells. Student's *t* test, unpaired, N=3 biological replicates. (**G**) LTBP1 RNA expression is associated with poor EGFR inhibitor response (CCLE database). (**H**) siRNA-mediated knock down of *FN1*, *ITGB3*, and *TGFβ2* affects EGFR expression. Hsp90 was used as a loading control. (I) CRISPR-mediated knockout of *FN1*, *ITGB3*, and *TGFβ2* affects EGFR expression. Hsp90 was used as a loading control. Experiments were conducted once in (**H and I**).

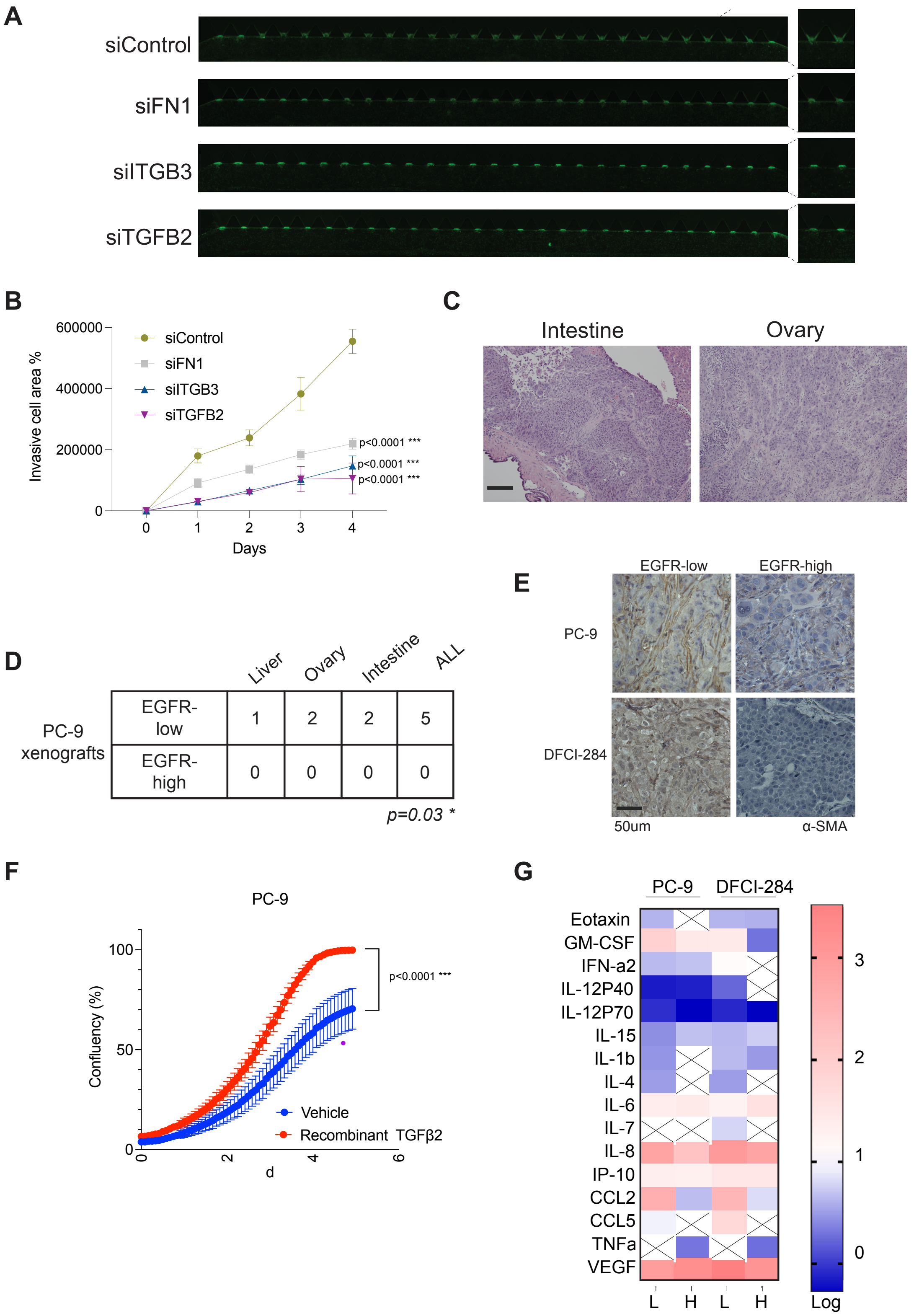

Supplementary Figure 5: (**A**) Images of siRNA knockdown of FN1, ITGB3, and TGFB2 in a microfluidic invasion assay: PC-9 EGFR-low cells were seeded into the proximal side channel of the microfluidic chip, where their invasion toward a collagen matrix was monitored over time. Serum was

added to the proximal side channel. (**B**) Quantification of the microfluidic invasion assay: ANOVA (one-way) was used for statistical analysis. N = 3 technical replicates. (**C**) Example H&E stainings from the metastatic tissues originating from the PC-9 EGFR-low xenograt mice. Scale bar is 1000 μm. (**D**) Sites of metastasis in PC-9 EGFR-low vs EGFR-high xenografted mice. N = 9 mice per group. Fisher's exact test. (**E**) Xenograft tumors grafted from EGFR-low cells are more positive for SMA and are enriched in fibers with SMA-expression. Scale bar is 50 μm. (**F**) Ectopic TGFβ2 is enhancing PC-9 cell line proliferation. Vehicle or 1 ng/ml recombinant TGFβ2 was added on the first day of the experiment. N = 1 biological replicates consisting of 6 technical replicates. (**G**) Cytokine profiling from the culture medium of EGFR-low (marked L) and EGFR-high (marked H) cells in PC-9 and DFCI-284. Medium was collected after 48 hours of culture. The PDXs experiments were conducted once in (**C, E**).

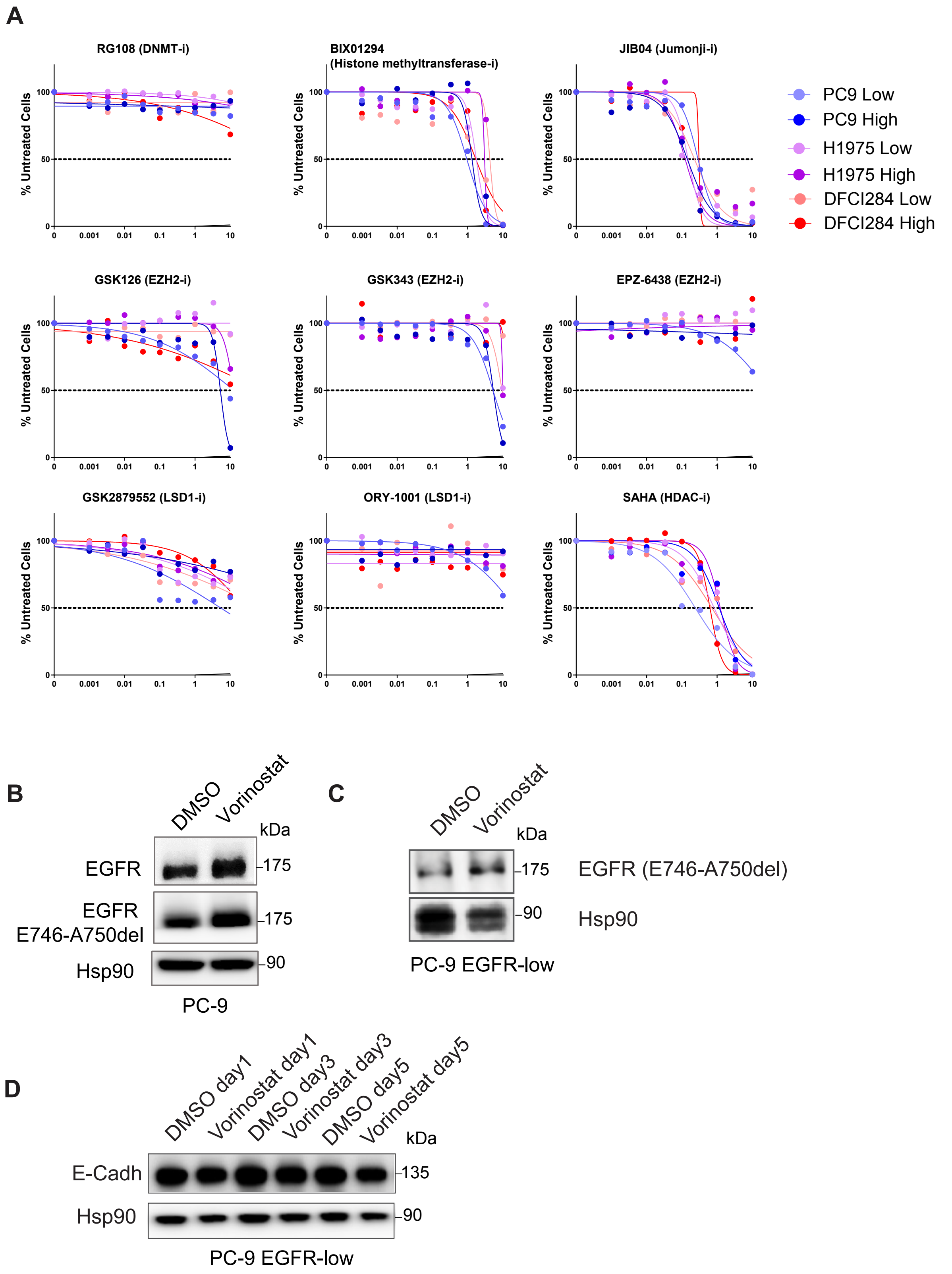

Supplementary Figure 6: (**A**) IC50-curves for epigenetic inhibitors used in the study. Cells were treated for 72 hours. (**B-C**) Vorinostat (SAHA) upregulates both total and mutant EGFR. PC-9 and PC-9 EGFR-low cells were treated with control DMSO or 100 nM vorinostat for 3 days, and blotted for total EGFR or EGFR exon19del-specific antibody. Hsp90 was used as a loading control. (**D**) E-Cahdherin expression in PC-9 EGFR-low cells treated with control DMSO or vorinostat for the

indicated amount of days. Hsp90 was used as a loading control. Depending on the marker, experiments were repeated one-three times in (**B, C, D**).

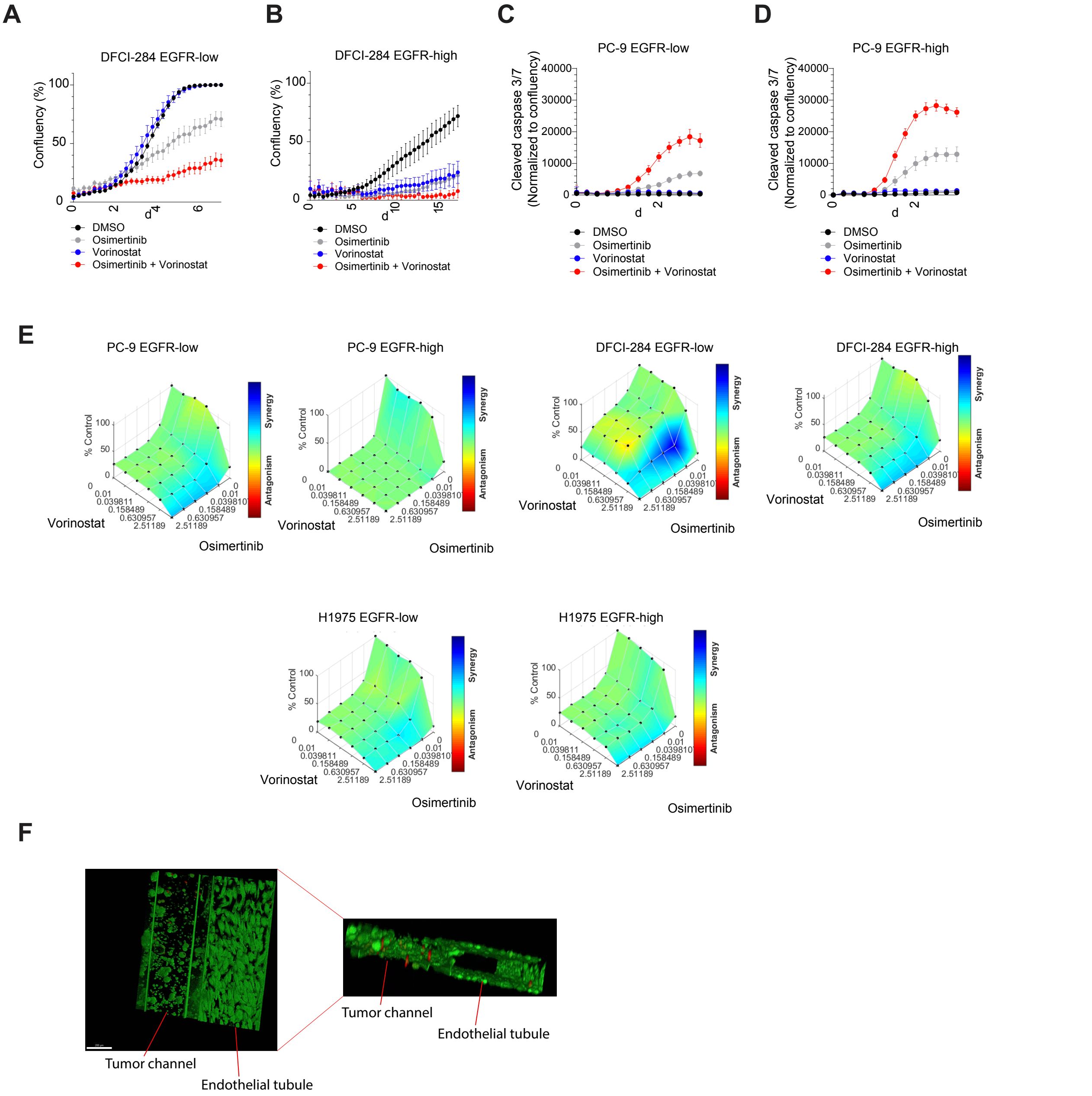

Supplementary Figure 7: (**A-B**) Combination efficacy of osimertinib + panobinostat combination in DFCI-284 EGFR-low vs EGFR-high cells. Cells were treated with either control vehicle, 10 nM Osimertinib, 1uM vorinostat or the combination of the two drugs. (**C-D**) PC-9 EGFR-low and EGFR-high cells are more apoptotic with Osimertinib + vorinostat combination than with the drug treatments alone. Cells were treated with either vechile, 10 nM osimertinib, 1uM vorinostat or the combination of the two drugs. (**E-G**) Synergy mapping of osimertinib + vorinostat in PC-9, DFCI-284 and H1975 EGFR-low vs EGFR-high cell lines. Blue color indicates drug synergy. N = 2 biologically independent experiments each consisting of 3 technical replicates. (**H**) Ectopic expression system for degradable mutant EGFR. Deg-GFP is a control plasmid that was used to test the degradation system. dTAG-13 compound was used to degrade the plasmids. (**I**) Ectopic mutant EGFR sensitizes EGFR-low cells to osimertinib, while its degradation reduces sensitivity. Cells were treated with vehicle DMSO, 10 nM osimertinib and/or 100 nM dTAG-13. (**J**) Introducing a stop codon before the degradation tag (only expressing mutant EGFR) reverses the sensitivity phenotype. (**F**) Endothelial tubule formation in the microfluidic device. HUVEC endothelial tubule was seeded in the distal right channel adjacent to the tumor channel in the middle (ECM). The tubule fully formed after 3 days, after which the cells were stained with Live-green/dead-red AO/PI staining and confocal images were acquired. Scale bar is 200 µm.

Supplementary Tables

Supplementary Table 3. Primer sequences used for the genomic PCR amplification of the TGFβ2 gRNA1 and gRNA2 targeted regions.

TGFβ2 gRNA1 and gRNA2 Genomic PCR primer Forward	TGFβ2 gRNA1 and gRNA2 Genomic PCR primer Reverse	Size of PCR fragment
1: GCAGCACACTCGATATGGAC<br>2: CCAGAAGACTATCCTGAGCC	1: TAGGGGGAAGGGAAAGATGG<br>2: GAGCAAAGAAAACAAACCTAC	1: 501 bp<br>2: 570 bp

Supplementary Table 4. Guide RNA sequences and vectors used for the CRISPR/ Cas9 knockout of FN1, and TGFβ2 in PC9 EGFR-low cells.

Gene	NCBI Gene ID	Target sequence_gRNA1	Target sequence_gRNA2	Vector name & Source
FN1	2335	ACTGACCCCCTTCATGGCAGCGG	-	pLenti-CRISPR-V2-GFP, Addgene # 82416
TGFβ2	7042	CGACGAAGAGTACTACGCCA	AGATGGAAATCACCTCCGGG	pLenti-CRISPR-V2-GFP, Addgene # 82416
Non-target		Lenti CRISPR Universal Non-Target Control #2, pLenti-CRISPR-V2-GFP, Addgene # 82416		pLenti-CRISPR-V2-GFP, Addgene # 82416

		CRISPR 19, Sigma-Aldrich		

Supplementary Table 5. Guide RNA sequences and vectors used for the CRISPR/ Cas9 knockout of ITGβ3.

Gene	NCBI Gene ID	Target sequence_sgRNA1	Target sequence_sgRNA2	Vector name & Source
ITGβ3	3690	TCACTCAAGTCAGTCCCCAG	GGTGAGCTTTCGCATCTGGG	pLenti-CRISPR-V2-GFP, Addgene # 82416
Non-target		GAGTGATGCTTAGACTCCGT	TTCGCACGATTGCACCTTGG	pLenti-CRISPR-V2-GFP, Addgene # 82416
