# Supplementary material for: Intratumor heterogeneity of EGFR expression mediates targeted therapy resistance and formation of drug tolerant microenvironment
Source: Nat Commun. 2025 Jan 2;16:28. doi: 10.1038/s41467-024-55378-5 (PMC11695629; doi:10.1038/s41467-024-55378-5)
Supplement: Supplementary file 4 — Description of additional Supplementary Files [file 41467_2024_55378_MOESM4_ESM.pdf]

Description of Additional Supplementary Files

Supplementary Data 1: EGFR staining heterogeneity and clinical features of the EGFRmut NSCLCs

Supplementary Data 2: Characteristics of the EGFR mutant lung cancer cell lines
