# Supplementary material for: Intratumor heterogeneity of EGFR expression mediates targeted therapy resistance and formation of drug tolerant microenvironment
Source: Nat Commun. 2025 Jan 2;16:28. doi: 10.1038/s41467-024-55378-5 (PMC11695629; doi:10.1038/s41467-024-55378-5)
Supplement: Supplementary file 5 — Reporting Summary [file 41467_2024_55378_MOESM5_ESM.pdf]

nature portfolio

Corresponding author(s): Heidi Haikala

Last updated by author(s): 30.9.2024

Reporting Summary

Nature Portfolio wishes to improve the reproducibility of the work that we publish. This form provides structure for consistency and transparency in reporting. For further information on Nature Portfolio policies, see our Editorial Policies and the Editorial Policy Checklist.

Statistics

For all statistical analyses, confirm that the following items are present in the figure legend, table legend, main text, or Methods section.

n/a	Confirmed	
☐	☒	The exact sample size (*n*) for each experimental group/condition, given as a discrete number and unit of measurement
☐	☒	A statement on whether measurements were taken from distinct samples or whether the same sample was measured repeatedly
☐	☒	The statistical test(s) used AND whether they are one- or two-sided<br>*Only common tests should be described solely by name; describe more complex techniques in the Methods section.*
☐	☒	A description of all covariates tested
☒	☐	A description of any assumptions or corrections, such as tests of normality and adjustment for multiple comparisons
☐	☒	A full description of the statistical parameters including central tendency (e.g. means) or other basic estimates (e.g. regression coefficient) AND variation (e.g. standard deviation) or associated estimates of uncertainty (e.g. confidence intervals)
☐	☒	For null hypothesis testing, the test statistic (e.g. *F*, *t*, *r*) with confidence intervals, effect sizes, degrees of freedom and *P* value noted<br>*Give P values as exact values whenever suitable.*
☒	☐	For Bayesian analysis, information on the choice of priors and Markov chain Monte Carlo settings
☒	☐	For hierarchical and complex designs, identification of the appropriate level for tests and full reporting of outcomes
☒	☐	Estimates of effect sizes (e.g. Cohen's *d*, Pearson's *r*), indicating how they were calculated

Our web collection on statistics for biologists contains articles on many of the points above.

Software and code

Policy information about availability of computer code

Data collection	Flow cytometry data was collected using Fortessa / Verse analyzers (BD) or FACSMelody when cells were also sorted (BD). Western blot images were acquired using Azumer 600 imager (Azure biosystems). Incucyte HD/Zoom/S5 or Cellcyte X were used for cell confluency analysis over time. Biospa (Biomek) and Cellcyte 5 were used for 3D cell culture analysis over time. Immunofluorescence images were taken using Nikon Eclipse 80i and Leica SP8 microscopes. IHC images were acquired using Leica DM LB or 3D Histech pannoramic 250 Flash III. Luminescence was measured using Spark multimode microplate reader.
Data analysis	Data was analyzed using Microsoft excel version 16.73 and analyzed & graphed using Graphpad Prism version 10.2.2 Images were analyzed using ImageJ version 1.53t-1.54g. Flow cytometry data was analyzed using FlowJo version 10.8.2.

For manuscripts utilizing custom algorithms or software that are central to the research but not yet described in published literature, software must be made available to editors and reviewers. We strongly encourage code deposition in a community repository (e.g. GitHub). See the Nature Portfolio guidelines for submitting code & software for further information.

Data

Policy information about availability of data

All manuscripts must include a data availability statement. This statement should provide the following information, where applicable:

- Accession codes, unique identifiers, or web links for publicly available datasets
- A description of any restrictions on data availability
- For clinical datasets or third party data, please ensure that the statement adheres to our policy

Representative data is provided in the manuscript and its supplementary files. Non-sensitive data generated or analyzed during this study, associated protocols and materials are provided by the corresponding author upon a reasonable request. RNA sequencing data from the cell lines is deposited in Gene Expression Omnibus (GEO) accession number GSE267515.

Research involving human participants, their data, or biological material

Policy information about studies with human participants or human data. See also policy information about sex, gender (identity/presentation), and sexual orientation and race, ethnicity and racism.

Reporting on sex and gender	The amount of treatment-naiive EGFRmut samples that we were able to acquire for the study was so low, that sex and gender were not taken into account in the patient selection.
Reporting on race, ethnicity, or other socially relevant groupings	This important point is denoted, however since the treatment-naiive patients were from Finland it has to be taken into account that the Finnish population is very homogeneous from the genetic features, so taking these factors into account is not very useful (small amount of representatives of ethnic/racial groups in a small subcategory of the patients).
Population characteristics	Patient-derived xenografts were generated from tumor biopsies or pleural effusions from EGFR mutant patients undergoing clinical biopsies and propagated in NSG mice. Presence of EGFR mutation in patient was detected using a gene panel.
Recruitment	The patient participation was voluntary and required an informed consent.
Ethics oversight	For treatment-naiive tumors, normal lung tissue and EGFRwt tumors used for histochemistry: A statement of approval for the study protocol was given by the Ethics Committee of the Hospital District of Helsinki and Uusimaa (HUS/970/2021) and research permit is from Helsinki University Hospital (HUS/237/2021).<br><br>For PDX Studies: Patient derived xenografts were generated from tumor biopsies or pleural effusions from EGFR mutant patients undergoing clinical biopsies and propagated in mice. All patients provided written informed consent. The study was conducted in accordance with the Declaration of Helsinki and was approved by the Dana Farber Cancer Institute.

Note that full information on the approval of the study protocol must also be provided in the manuscript.

Field-specific reporting

Please select the one below that is the best fit for your research. If you are not sure, read the appropriate sections before making your selection.

[x] Life sciences [ ] Behavioural & social sciences [ ] Ecological, evolutionary & environmental sciences

For a reference copy of the document with all sections, see nature.com/documents/nr-reporting-summary-flat.pdf

Life sciences study design

All studies must disclose on these points even when the disclosure is negative.

Sample size	In an experiment at least 3 biological replicates were included for cell based assays and each experiment was repeated at least 3 times. For in vivo mice experiments no statistical methods were used to pre-determine sample sizes but our sample sizes are similar to those reported in previous publications. REF: Eser PO, Paranal RM, Son J, Ivanova E, Kuang Y, Haikala HM, et al. Oncogenic switch and single-agent MET inhibitor sensitivity in a subset of EGFR-mutant lung cancer. Sci Transl Med. 2021;13(609):eabb3738.
Data exclusions	In vivo experiment: Results from one mouse was removed in Osimertinib group and one mouse from Osimertinib + Panobinostat group were removed due to the tumors developing alcers and had to be removed from the study ahead of time. The same mice were removed from the whole experiment including both tumor growth and FLI/BLI analysis. The NK cell killing assay in Figure 5J was biologically replicated only twice due to a shortage in NK cells caused by a change in an agreement in receiving them. In Fig 6K one value was excluded since it was significantly lower than others. For tumor-on-chip data 3-5 technical replicate chips were used per each condition. For longitudinal analysis of confluency (%), experiments were repeated at least 3 times, but only one representative with replicates is shown in the figure due to slight changes in each experiment caused by minor seeding confluency variation affecting the growth of the cells over time. However all the biological replicates were showing same results so this practice was accepted.

Replication	At least 3 biological replicates were used for experiments.
Randomization	For in vivo experiments: Tumors were allowed to establish to 200 ± 50 mm3 in size before randomization using Studylog software (San Francisco, CA) into various treatment groups with 8-10 mice per group.
Blinding	For histochemical stainings the slides were blinded from the researchers when they were derived from a cancer patient, but not when they were derived from a mouse. For ex vivo experiments data collection and analysis were not performed blind to the conditions of the experiments due to practical reasons.

Reporting for specific materials, systems and methods

We require information from authors about some types of materials, experimental systems and methods used in many studies. Here, indicate whether each material, system or method listed is relevant to your study. If you are not sure if a list item applies to your research, read the appropriate section before selecting a response.

Materials & experimental systems

n/a	Involved in the study
☐	☒ Antibodies
☐	☒ Eukaryotic cell lines
☒	☐ Palaeontology and archaeology
☐	☒ Animals and other organisms
☒	☐ Clinical data
☒	☐ Dual use research of concern
☒	☐ Plants

Methods

n/a	Involved in the study
☒	☐ ChIP-seq
☐	☒ Flow cytometry
☒	☐ MRI-based neuroimaging

Antibodies

Antibodies used	EGFR SP84 (#MA5-16360, Thermo Fisher), EGFR E746-A750del specific (D6B6, XP #2085 Cell Signaling Technology, CST), HSP90 (# #SC-794, Santa Cruz Biotechnology), E cadherin (# 3195S, CST), SMA, BIM (#2933 CST), HDAC1 (# 5356S, CST), HDAC2 (# 5113S, CST), HDAC3 (# 3949S, CST), HDAC4 (#15164, CST), HDAC6 (#7558, CST), H3-AC, GAPDH (# 6515, CST). Selected antibodies were validated by the vendors and were selected based on the number of publications. All antibodies were also tested in the lab (see validation below).
Validation	For immunohistochemistry multiple antibodies were tested in the beginning including controls and negative controls performed within the validation. Each antibody was tested with multiple different concentrations and the best performing antibody was selected for the actual study. For flow cytometry each antibody was compared with non-stained and IgG control, and typically the antibodies were titrated in the beginning to find the best concentration for flow. For western blot antibodies expressing the presence of only the appropriate bands that were right size (as published in the literature) was used.

Eukaryotic cell lines

Policy information about cell lines and Sex and Gender in Research

Cell line source(s)	HCC4006 and H1975 were from ATCC. PC-9 were from Dr. Kazuto Nishio (Kindai University, Osaka, Japan). DFCI-284, DFCI-243 and DFCI-169 were established in the Jänne Laboratory / Dana-Farber Cancer Institute.
Authentication	Commercially available cell lines (PC-9, HCC4006, H-1975) were DNA fingerprinted, for in-house primary cell lines presence of the driver mutation was confirmed using ddPCR.
Mycoplasma contamination	Cells were routinely tested against Mycoplasma contamination using PCR-based testing or MycoALERT Plus Mycoplasma detection kit (Lonza).
Commonly misidentified lines (See ICLAC register)	None of the cell lines used were found from the list of commonly misidentified lines.

Animals and other research organisms

Policy information about studies involving animals; ARRIVE guidelines recommended for reporting animal research, and Sex and Gender in Research

Laboratory animals	For PC9 xenograft model, female NCr nude mice, 6-weeks old were purchased from Taconic Bioscience, Inc. (Germantown, NY). Animals were acclimated for at least 5 days before initiation of study. Patient-derived xenografts were generated from tumor biopsies or pleural effusions from EGFR mutant patients undergoing clinical biopsies and propagated in NSG mice as described previously. REF: Eser PO, Paranal RM, Son J, Ivanova E, Kuang Y, Haikala HM, et al. Oncogenic switch and single-agent MET inhibitor sensitivity in a subset of EGFR-mutant lung cancer. Sci Transl Med. 2021;13(609):eabb3738.

Wild animals	Study did not involve wild animals.
Reporting on sex	Sex was not considered in the study design. Female mice were used in the study.
Field-collected samples	Study did not involve field-collected samples.
Ethics oversight	All animal studies were conducted at Dana-Farber Cancer Institute with the approval of the Institutional Animal Care and Use Committee in an AAALAC accredited vivarium.

Note that full information on the approval of the study protocol must also be provided in the manuscript.

Flow Cytometry

Plots

Confirm that:

- [x] The axis labels state the marker and fluorochrome used (e.g. CD4-FITC).
- [x] The axis scales are clearly visible. Include numbers along axes only for bottom left plot of group (a 'group' is an analysis of identical markers).
- [ ] All plots are contour plots with outliers or pseudocolor plots.
- [x] A numerical value for number of cells or percentage (with statistics) is provided.

Methodology

Sample preparation	Flow cytometry for cultured cell lines:<br><br>Cells were collected using Accutase (A1110501, Thermo Fisher Scientific), and counted with Countess cell counter (Invitrogen). Suspension confluency was adjusted to 1x106 cells / ml, after which the cells were stained with 1:100 zombie viability dye for 20 min in the dark at RT. Cells were spun down and washed with PBS, followed by 30 min staining with conjugated antibodies in flow buffer (10 % FBS-PBS), on ice and in the dark. Samples were spin washed 3 times, resuspended into flow buffer and acquired using Fortessa analyzer (BD Biosciences). The data were analyzed with FlowJo version 10.8 (BD Biosciences). Cell sorting was conducted using a BD Melody instrument.<br><br>Flow cytometry for patient-derived xenograft tumors:<br><br>Patient derived xenograft tumors were collected and minced with a scalpel, after which they were shaken at 140 RPM in 0.2 % collagenase A and cell culture medium for 3-4 hrs, +37 °C. The cell suspension was treated with TrypLe Express solution (12604013, Thermo Fisher Scientific) for 20 minutes at +37 °C to obtain a single cell suspension. The cell suspension was adjusted to 1x106 cells / ml and stained with zombie viability dye (BioLegend) for 20 min at RT in the dark. Cells were spun down and washed with PBS, followed by 30 min staining with conjugated antibodies in flow buffer (10 % FBS-PBS), on ice and in the dark. Samples were spin washed 3 times, resuspended into flow buffer and acquired using Fortessa analyzer (BD). The data were analyzed with FlowJo version 10.8 (BD Biosciences).
Instrument	Analysis was performed using Fortessa analyzer (BD). Sorting was performed using FACSMelody (BD).
Software	Analysis was performed using FlowJo 10.8.2.
Cell population abundance	Minimum 100 000 cells were sorted for each tube to avoid selection.
Gating strategy	For flow analysis: FCS-A/SCS-A was used for selecting single cells, after which zombie viability markers (BioLegend) were used to take out the dead cells from the analysis. IgG control and unstained sample were used for determining the positive and negative populations. For sorting both SSC-A/FSC-A and then SSC-A/SSC-H were used to determine singlet cells. Unlike in the shown figure to illustrate the EGFR low/high population, the EGFR sorting population was chosen to be uniform in size scatter (diagonal selection of cells rather than just a "box").

- [x] Tick this box to confirm that a figure exemplifying the gating strategy is provided in the Supplementary Information.
